# Trait Analysis in Domestic Rabbits (*Oryctolagus cuniculus f. domesticus)* Using SNP Markers from Genotyping-by-Sequencing Data

**DOI:** 10.3390/ani12162052

**Published:** 2022-08-11

**Authors:** Congyan Li, Yuying Li, Jie Zheng, Zhiqiang Guo, Xiuli Mei, Min Lei, Yongjun Ren, Xiangyu Zhang, Cuixia Zhang, Chao Yang, Li Tang, Yang Ji, Rui Yang, Jifeng Yu, Xiaohong Xie, Liangde Kuang

**Affiliations:** 1 Animal Breeding and Genetics Key Laboratory of Sichuan Province, Sichuan Animal Science Academy, Chengdu 610066, China; 2 Sichuan Academy of Agricultural Sciences, Chengdu 610066, China

**Keywords:** genotyping-by-sequencing, single-nucleotide polymorphism, domestic rabbits

## Abstract

**Simple Summary:**

Rabbit breeding is an important branch of agricultural animal breeding; their fur color and weight are desirable traits for artificial breeding. Polymorphism can provide potential molecular markers for studying rabbit traits and improve rabbit breeds with such markers in the future. In this study, single-nucleotide polymorphism markers in genotyping-by-sequencing data were used to analyze rabbit traits. In total, three genes were identified to be associated with fur color and four with weight. The results of this study provide a data base for the research and improvement of rabbit breeding program.

**Abstract:**

The domestic rabbit (*Oryctolagus cuniculus f. domesticus*) is a very important variety in biomedical research and agricultural animal breeding. Due to the different geographical areas in which rabbit breeds originated, and the long history of domestication/artificial breeding, rabbits have experienced strong selection pressure, which has shaped many traits of most rabbit varieties, such as color and weight. An efficient genome-wide single-nucleotide polymorphism (SNP) detection strategy is genotyping-by-sequencing (GBS), which has been widely used in many organisms. This study attempted to explore bi-allelic SNPs associated with fur color and weight-related traits using GBS in five rabbit breeds. The data consisted of a total 831,035 SNPs in 150 individuals from Californian rabbits (CF), German Zika rabbits (ZK), Qixing rabbits (QX), Sichuan grey rabbits (SG), and Sichuan white rabbits (SW). In addition, these five breeds of rabbits were obviously independent populations, with high genetic differentiation among breeds and low genetic diversity within breeds. A total of 32,144 SNP sites were identified by selective sweep among the different varieties. The genes that carried SNP loci in these selected regions were related to important traits (fur color and weight) and signal pathways, such as the MAPK/ERK signaling pathway and the Hippo signaling pathway. In addition, genes related to fur color and weight were identified, such as *ASIPs*, *MITFs* and *KITs, ADCY3s*, *YAPs*, *FASs*, and *ACSL5s*, and they had more SNP sites. The research offers the foundation for further exploration of molecular genetic markers of SNPs that are related to traits.

## 1. Introduction

Domestic rabbits (*Oryctolagus cuniculus f. domesticus*) are very important varieties for biomedical research and agricultural animal breeding because they are mild and easy to handle, restrict and reproduce [1]. At present, there are many different breeds of rabbits due to regional differences and artificial selection. According to body size, rabbits are generally divided into large rabbits, medium rabbits and small rabbits. For instance, Sichuan grey rabbits (SG) and Sichuan white rabbits (SW) are small breeds with a slow growth rate [2]. China has more than 20 indigenous and imported rabbit breeds, which are used to produce meat and fur [3]. Although the production performance of local rabbit breeds in China is generally lower than that of imported rabbit breeds, indigenous breeds have advantages in disease resistance and meat quality. China is the world’s largest country for rabbit-meat consumption and production [4], so improving the production capacity of local varieties is crucial to the sustainable development of the Chinese rabbit industry. Unfortunately, the study of the genetic diversity and characteristic genes of Chinese indigenous rabbits is not deep enough.

Nowadays, due to the development of high-throughput genotyping technology, it is possible to study the genetic diversity of species and to distinguish and select different breeds of rabbits by identifying single-nucleotide polymorphism (SNP) markers. The great advantages of SNPs as genetic markers is that they have a relatively low mutation rate and are distributed on all chromosomes [5]. Carneiro et al. [6] reported that wild and domestic rabbits evolved via allelic frequency changes at many loci, which carry many SNPs. However, it is still unclear how to distinguish the traits of different rabbit breeds according to SNP markers.

Genotyping-by-sequencing (GBS) can acquire the whole genome variation information of a population composed of hundreds of individuals, aiming to shorten the complexity of the genome to be sequenced [7]. The GBS method is a cost-effective alternative to the existing genotyping methods, in which the SNPs detected by the GBS method can be successfully applied to population genetics to reveal the genetic differentiation of the studied varieties [8,9].

In this study, in order to explore the genetic diversity and population structure of indigenous breeds and other breeds, five breeds of rabbits were selected as experimental subjects to perform GBS, the California rabbit (CF), German Zika rabbit (ZK), Qixing rabbit (QX), SG and SW. We assessed the number, distribution and diversity of SNPs detected. Our aim was to identify SNP markers among the five breeds of rabbits to identify genetic changes in rabbit traits among different breeds.

## 2. Methods and Materials

### 2.1. Sample Information

QX is a new breed of rabbit, which is of medium size, fast growth, high yield and strong disease resistance [10]. ZK has the advantages of larger body size, faster growth and greater daily gain [11]. CF is a medium-sized variety with good reproductive performance and growth rates similar to QX [12]. The study included 30 QX rabbits from Sichuan Animal Science Academy in Chengdu, SG and SW rabbits from Sichuan, and CF and ZK samples from overseas regions (n = 30). CF and SG are colored rabbits, while the others are white. SW and SG are small breeds with a slow growth rate, CF and QX are medium breeds with a relatively fast growth rate, and ZK is a large breed of rabbit that grows the fastest. Samples were taken from male and female individuals. In this study, genomic DNA was extracted from whole blood. All animals were kept in a pathogen-free environment and fed ad lib. The procedures for care and use of animals were approved by the Ethics Committee of the Sichuan Animal Science Academy (No. 20210112) and all applicable institutional and governmental regulations concerning the ethical use of animals were followed.

### 2.2. Library Construction and Sequencing

The libraries were prepared by following the protocol developed by Qi et al. (2018). Genomic DNA was extracted from whole blood using GenElute blood genomic DNA kit (NA2010-1KT; Sigma-Aldrich, Shanghai, China). After having assessed DNA sample quantity and quality by NanoDrop 2000, the qualifying DNA samples were used for library construction using the SuperGBS technology [13]. The restriction enzyme PstI-HF/MspI (R3140/RO106; New England BioLabs, Beijing, China) was utilized to digest genomic DNA (200 ng), and each end of the digestion products was ligated with barcoded adapter using T4 DNA ligase (M0202; New England BioLabs, Beijing, China). Fragments of 300–700 bp were purified and recovered by a modified magnetic bead recovery system of Sera-Mag SpeedBeads (65152105050250; GE Healthcare Life Sciences). After that, fragments containing adapters on both ends were amplified using PCR. Purified sample libraries were quantified using Qubit dsDNA HS Assay Kit (Q3258; Invitrogen, Carlsbad, CA, USA), and concentrations lower than 5 ng/μL were eliminated. Thirty nanograms of each GBS library were pooled. Pooled GBS libraries (30 samples per species; 900 ng per library) were subjected to pair-end sequencing (150 bp) run on NovaSeq 6000 platform (Illumina, San Diego, CA, USA).

### 2.3. Data Quality Control and SNP Calling

Raw reads were split from the down-machine data according to the barcode and restriction site information using the Stacks software [14] with option –r --renz _1 --adapter_mm 1, and then qualified by FastQC software [15] to obtain clean reads. Next, clean reads were aligned to the rabbit reference genome (GCA_000003625) using BWA software (v0.7.17) [16] mem with default parameters. Genome Analysis Toolkit (GATK, v4.1.3) [17] was used to call out all the variants, including SNPs and Indels. To obtain robust results in the subsequent analyses, the following criteria were applied for variant filtering using VCFtools (v0.1.16) [18]. (1) Loci with sequencing depth < 4 were removed. (2) SNPs with a minor allele frequency (MAF) < 0.01 were discarded. (3) SNPs that were missing in more than 20% of the samples were removed. Then SnpEff (v4.1g) [19] was applied to predict (potential) functional of the filtered bi-allelic of SNPs.

### 2.4. Phylogenetic Tree and Principal Component Analysis (PCA)

The R package ggtree (v1.16.6) was used for visualizing the phylogenetic tree. PCA of the SNPs was performed using genome-wide complex trait analysis (GCTA) software (v1.26.0) [20].

### 2.5. Genetic Diversity

The diversity parameters such as expected heterozygosity (He), polymorphism information content (PIC), nucleotide diversity (Pi), observed heterozygosity (Ho), and coefficient of gene differentiation (Fst) were calculated for each SNP marker using VCFtools (v0.1.16) [18].

### 2.6. Selective Sweep

The selective sweep analysis methods such as Fst, Pi, and joint analysis of Fst and Pi were calculated for each SNP marker. GO and KEGG enrichment were performed using the genes where the SNP sites of the selected regions were located and the sites not located in the gene body region (e.g., upstream, downstream, etc.) were removed.

### 2.7. Quality Control

A total of 150 samples were collected in this project and divided into 5 groups (30 individuals per group). The number of clean bases obtained in each individual was between 1.26 gigabases (Gb)–2.06 Gb, and the ratio of clean base to raw base ranged from 93.88–97.25%; the number of clean reads was between 0.00863–0.0141 Gb, and the ratio of clean reads to raw reads was in the range of 94.07–97.58%; the range of Q30 was 89.44–91.34% (Supplementary Table S1). Moreover, in this study, the mapping ratio in each individual was between 98.51–98.86%, and SNPs with coverage ≥1× ranged from 7.14 to 10.37% (Supplementary Table S2). These results indicated that the sequencing quality was good and could be used for further analysis.

## 3. Results

### 3.1. GBS-Based Genome-Wide Discovery of SNPs

**Figure 1:**
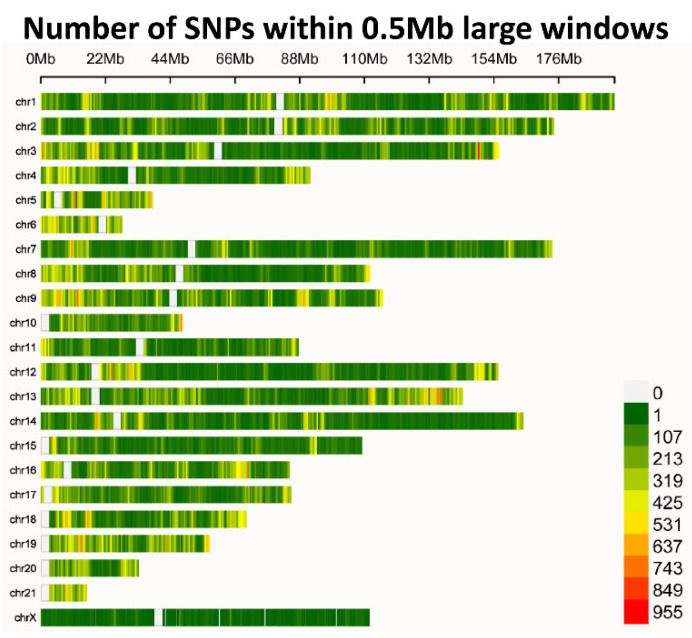
Distribution of SNPs along chromosomes. The vertical axis displays chromosomes (sorted by number) and the horizontal axis represents size of chromosomes. The color bar to the right indicates number of SNPs detected per window.

Based on clean reads, a total of 831,035 SNPs were left after GATK identification and applying filtering control by VCFtools. These high-quality SNPs were used for further analysis. The number of SNPs on chromosome 6 and 21 was relatively small (Figure 1). The SNP density reached up to 1 variant every 3279 bases (Supplementary Table S3). Among the 831,035 SNPs, transitions occurred more frequently with A/G (37.1%, 308,583 SNPs) being almost equal to C/T (37.2%, 309,196 SNPs) (Supplementary Table S4). Among transversions, a similar phenomenon was also observed, except that the frequency of A/T was significantly lower, the frequency of the other three types of base substitution was almost the same. We detected 256 SNP variants that may have a high degree of influence on the protein, possibly resulting in protein truncation or loss of function (Supplementary Table S5).

### 3.2. Population Structure

**Figure 2:**
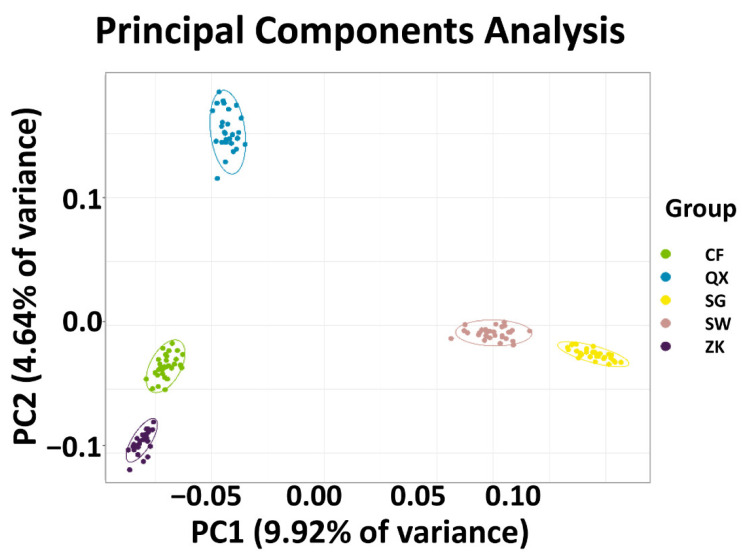
PCA based on 831,035 SNPs detected across all five rabbit breeds. CF, Californian rabbits; ZK, German Zika rabbits; QX, Qixing rabbits; SG, Sichuan grey rabbits; SW, Sichuan white rabbits.

In order to further understand the genetic structure of the rabbit breeds, we used population genomics tools for analysis. The PCA, applied to study the genetic relationship among breeds, revealed a close relationship between CF and ZK and as expected also between SW and SG (Figure 2). Specifically, the genetic relationship between CF and ZK was close, while that between SW and SG was close. Interestingly, SW and SG were only differentiated on PC1, possibly due to the fact that both are small cultivars with different colors. Therefore, SNPs in principal component PC1 may be correlated with color, which causes the difference between SW and SG. In addition, PC2 has the largest breed (ZK) at the bottom, so maybe PC2 has weight-related SNPs. CF and QX were medium-sized breeds, so they may be different from ZK due to weight-related SNPs. Taken together, these findings proved that SNP markers can well distinguish five different breeds of rabbit, and the breeds from the same region are more closely related.

### 3.3. Genetic Diversity

The European rabbit (*Oryctolagus cuniculus*) is the only recognized ancestor of the domestic rabbit. Therefore, we chose wild rabbits from France as a comparison to investigate to what degree the breeding process had reduced genetic variability. Regarding the value of He, French wild rabbits showed the highest levels of genetic diversity (He = 0.723) [21], followed by CF (He = 0.25), QX (He = 0.25), SW (He = 0.24), ZK (He = 0.24), and finally SG (He = 0.21) (Table 1), which indicated the genetic diversity of the five breeds was poor. Furthermore, the value of PIC in each breed ranged from 0.17 to 0.20; Pi value was 0.24–0.26; Ho value was 0.20–0.24. On the whole, these five breeds had poor genetic diversity, and there was not much difference between them. In addition, Fst values were used to indicate whether genetic differentiation existed among subpopulations. For example, the genetic differentiation between ZK and SW was most pronounced compared to SW vs. other breeds (Supplementary Table S6). Taken together, it was found that each breed showed genetic balance, and there was genetic differentiation among different breeds.

### 3.4. Identifying SNP Markers Associated with Trait Genes

**Figure 3:**
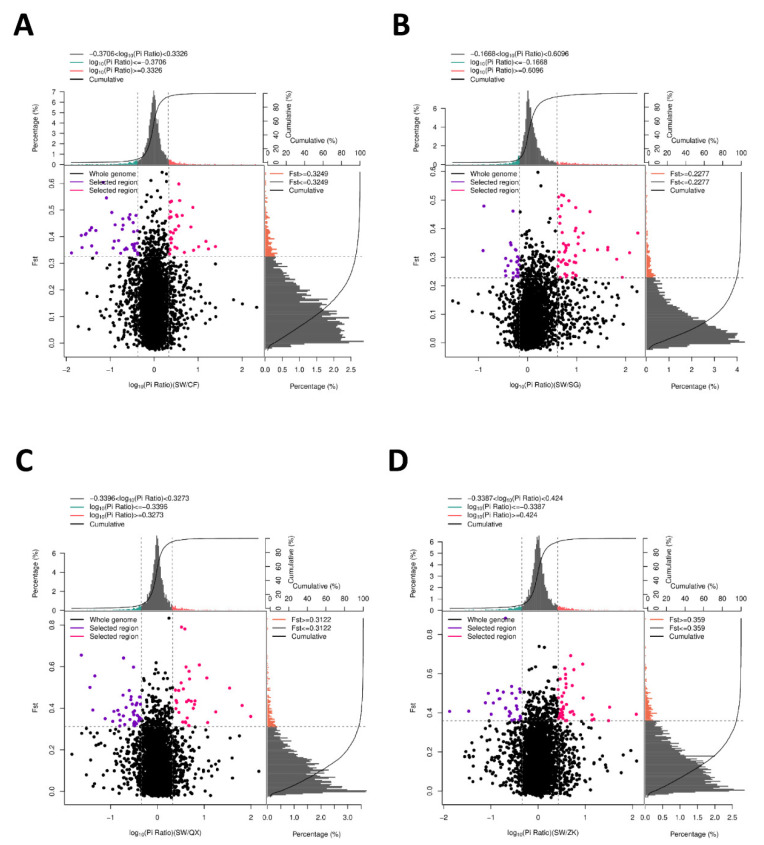
Selective elimination analysis of Fst and Pi between breeds. (**A**) The selective elimination analysis of Fst and Pi between SW and CF. (**B**) The selective elimination analysis of Fst and Pi between SW and SG. (**C**) The selective elimination analysis of Fst and Pi between SW and QX. (**D**) The selective elimination analysis of Fst and Pi between SW and ZK. The horizontal axis represents Pi ratio (Log10) value, the vertical axis represents Fst value, and the point plot in the middle represents the corresponding RATIO of Fst to Pi in different Windows. The top green and red region is the top 5% region selected by Pi, the right orange region is the top 5% region selected by Fst, and the middle red and purple region is the intersection of Fst and Pi, which is SNP loci in the selective eliminated region.

We further analyzed the selective sweep among the breeds and mined the selected genes in the different traits (fur color and weight) of the breeds. In view of this, we used SW as the control to screen the selected genes controlling fur color (SW vs. SG; SW vs. CF) and weight (SW vs. QX; SW vs. ZK) compared with the other groups. First, according to the empirical distribution of Fst (Supplementary Figure S1), candidate regions under selection were defined as outliers falling with the upper 5% of the distribution of Fst. The area where the red and purple areas intersect was the intersection of Fst and Pi, which is the candidate region (1 Mb window size) (Figure 3). The results showed that there was obvious genetic differentiation between these groups.

**Figure 4:**
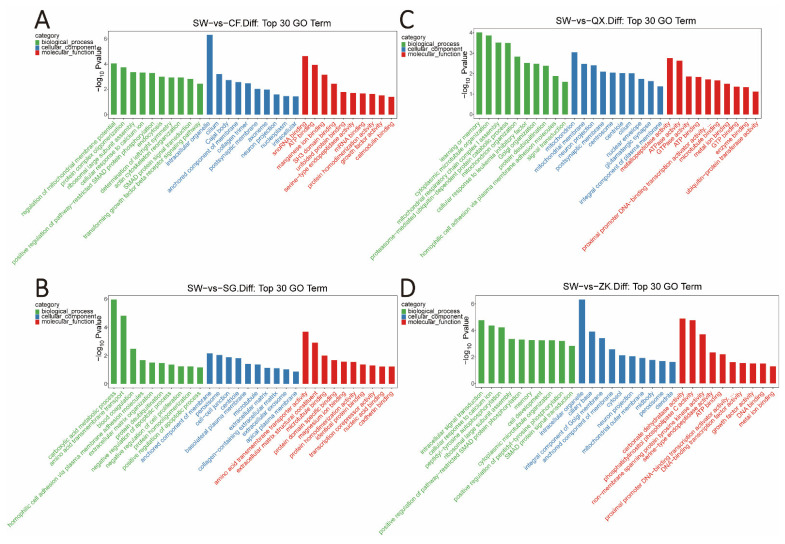
GO enrichment analysis was performed for genes containing SNPs identified by outlier analysis. (**A**) Top 30 GO terms for SW and CF. (**B**) Top 30 GO terms for SW and SG. (**C**) Top 30 GO terms for SW and QX. (**D**) Top 30 GO terms for SW and ZK.

**Figure 5:**
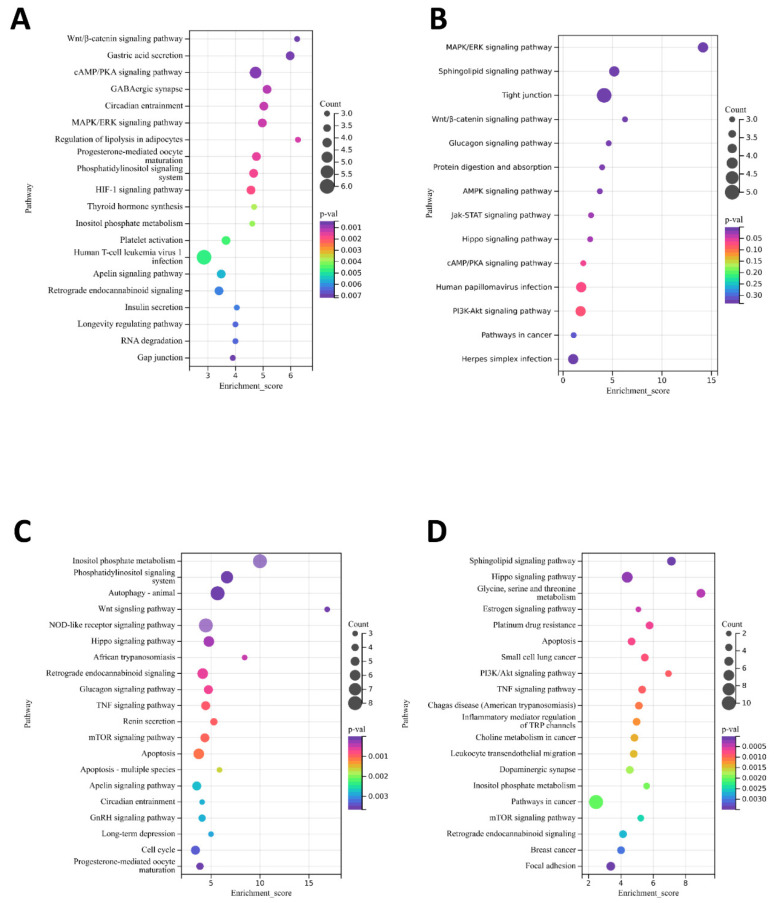
KEGG pathways enrichment analysis was performed for genes with SNP loci in the selected region. (**A**) KEGG pathways enrichment analysis for SW and CF. (**B**) KEGG pathways enrichment analysis for SW and SG. (**C**) KEGG pathways enrichment analysis for SW and QX. (**D**) KEGG pathways enrichment analysis for SW and ZK.

Furthermore, genes with 32,144 SNP loci in the selective eliminated region were functionally enriched to screen out genes controlling specific functional traits. Most of these genes were involved in protein synthesis and cell proliferation in a variety of cellular and biochemical processes (Figure 4 and Figure 5). By further comparison of SW vs. SG and SW vs. CF, we found that they were enriched in signaling pathways related to fur color [22,23,24], including the MAPK/ERK signaling pathway, cAMP/PKA signaling pathway and Wnt/β-catenin signaling pathway (Figure 5A,B). In the above pathways, we identified three known genes (*ASIPs*, *MITFs* and *KITs*) associated with fur color [25,26], which had more SNPs site (Supplementary Table S7). In addition, in the weight group (SW vs. QX; SW vs. ZK), they were co-enriched in the signaling pathway related to weight [27,28], such as the Hippo signaling pathway and the mTOR signaling pathway (Figure 5C,D). Similarly, we found genes (*ADCY3s*, *YAPs*, *FASs*, and *ACSL5s*) related to weight in these pathways [29,30], and their SNPs are shown in Supplementary Table S8. Collectively, the genetic differentiation between the different breeds is large, and the genes containing SNPs loci in the selective eliminated region have the function of regulating traits.

## 4. Discussion

Rabbits can be divided into large, medium and small according to their body size. At present, the growth speed and body size of rabbits have an impact on meat quality [31]. However, there are few studies on the genetic diversity of rabbits. In the present study, we used GBS to analyze the SNPs of five varieties of rabbits. The results showed that the rabbits of the five varieties showed independent populations and could be distinguished by SNP markers. Therefore, we decided to use the GBS method to screen out the genes that control rabbit phenotypic traits and improve our understanding of rabbit differentiation at the molecular level.

GBS is a genetic screening technology to find SNPs and genotype them. Sequence-based genotyping provides a low-cost alternative microarray for the study of genetic variation [32]. Pértille and his colleagues studied bird breeds’ genomics using a GBS approach [33]. Our goal was to detect SNPs to analyze the genetic diversity of different rabbit breeds, and to screen the genes related to specific functional traits through functional enrichment analysis of the selected SNP loci.

We obtained a total of 831,035 SNPs in the markers panel through screening. Compared with other GBS studies, our analysis allowed the identification of relatively more SNPS, such as ducks (169,209) [34]. In addition, compared with the phylogenetic analysis of the same material using microsatellite marker methods that cannot identify the offspring of different species so clearly [35], our GBS method has obvious advantages, which can clearly distinguish between different breeds of rabbit. Furthermore, in comparison with French wild rabbits [21], the genetic diversity of the rabbits in these five breeds was poor. We further found that these rabbits had obvious genetic differentiation. Similar to other studies [36], this may be due to genetic isolation and low gene flow caused by geographical distance.

Advances in genomic selection technology and the shortening of the generation interval make it an effective method for animal breeding. Through selective scanning analysis, 39 common chromosomal regions and 102 protein-coding genes were found to be affected in broilers, and these genes were enriched in tight junction pathways and myosin complexes, which regulate meat production and quality [37]. In our study, we also performed GO and KEGG enrichment analysis on genes with SNP loci in the selective eliminated region to explore the genes that control fur color and weight. We found that these genes are involved in biological functions such as protein synthesis and cell development. Moreover, color-related groups (SW vs. SG; SW vs. CF) were mainly enriched in MAPK/ERK signaling Pathway, cAMP/PKA signaling Pathway, and Wnt/β-catenin signaling Pathway, which are common signaling pathways related to fur color [38]. Liu et al. [39] reported that the MAPK/ERK pathway plays an important role in inhibiting melanin production. We looked for genes associated with fur color along these pathways, eventually finding *ASIPs*, *MITFs* and *KITs*. Mammalian coat color is mainly associated with the pigment distribution or proportion of eumelanin and brown melanin [40], in which MC1R and ASIP regulates the relative ratio of eumelanin to melanin [41]. In addition, some reported the SNP s66432.1 in ASIP was significantly associated with white and non-white coat changes [25]. However, we did not find the strongest signal about SNPs in ASIP, but we found 20 SNPs in ASIP, which may explain the fur color variation from grey to white. Furthermore, variations in the *MITF* gene result in defective melanin transport, resulting in diluted coat color in mammals [42]. In our study, there were 14 variants in rabbit *MITF* gene, which may be related to rabbit coat color.

In addition, we also identified genes related to weight (SW vs. QX; SW vs. ZK), such as *ADCY3s*, *YAPs*, *FASs*, and *ACSL5s*. Grarup et al. [43] reported that loss of function in *ADCY3s* increases the risk of obesity and diabetes. SNP rs2419621 (C>T), which is common in the *ACSL5s* promoter, is associated with weight loss in obese white women [44]. In the study, 2 SNPs (c.1477-57C>T, c.1477-47A>G) in three rabbit breeds (SW, QX and ZK) were identified in the *ACSL5s* gene, which may influence weight changes. Furthermore, according to the functional annotation of candidate genes, several pathways are directly related to the growth and development of animals. For instance, the Hippo signaling pathway is a pathway that inhibits cell growth [27]. The MTOR pathway is involved in cellular autonomic nutrition sensing, affecting animal fat metabolism and regulating animal growth and maturation [45]. Taken together, some SNPs in these genes may influence the formation of fur color and weight in rabbits, which may lead to different degrees of genetic differentiation.

## 5. Conclusions

This study showed that GBS is an effective marker development technique for rabbit breed isolation and genetic studies. We found that PCA by SNP markers supported the segregation of different breeds. Similarly, the genetic differentiation among the five breeds was significant, and the genetic diversity was poor. Some genes with SNP loci may be related to the weight and color of rabbit breeds, such as *ASIPs*, *MITFs* and *KITs, ADCY3s*, *YAPs*, *FASs*, and *ACSL5s*. Our results provide a theoretical reference for marker-assisted selection breeding for genetic improvement programs in rabbits.

## Figures and Tables

**Table 1:** Summary of F-statistics for all rabbit breeds.

Breeds	He	PIC	Pi	Ho
Californian rabbits	0.25	0.20	0.25	0.24
Sichuan white rabbits	0.24	0.20	0.25	0.23
Sichuan grey rabbits	0.21	0.17	0.21	0.20
German Zika rabbits	0.24	0.19	0.24	0.23
Qixing rabbits	0.25	0.20	0.26	0.24

Notes: He, expected heterozygosity; PIC, polymorphism information content; Pi, nucleotide diversity; Ho, observed heterozygosity.

## Data Availability

We have uploaded all original data to the NCBI database (PRJNA832894), and the data that support the findings of this study are openly available at https://www.ncbi.nlm.nih.gov/sra/docs/submitupdate/.

## Supplementary Materials

The following supporting information can be downloaded at: https://www.mdpi.com/article/10.3390/ani12162052/s1, Supplementary Table S1. Quality control statistics for GBS. Supplementary Table S2. Mapping rate statistics for GBS sequencing. Supplementary Table S3. Information of base variation in SNP. Supplementary Table S4. Summary results of nucleotide substitution types at SNP loci. Supplementary Table S5. Summary results of the potential functional effect of SNP variants on gene products. Supplementary Table S6. Pairwise comparison of genetic differentiation and genetic distances among rabbit breeds. Supplementary Table S7. Genes associated with fur color carry information about SNPs. Supplementary Table S8. Genes associated with weight carry information about SNPs. Supplementary Figure S1. Coefficient (Fst) of genetic differentiation between breeds. The abscissa represents different chromosome names, the ordinate represents the Fst value in the corresponding chromosome window, and the two dotted lines represent the two selection thresholds (top 5% or 1%).
