# Supplementary material for: Trait Analysis in Domestic Rabbits (Oryctolagus cuniculus f. domesticus) Using SNP Markers from Genotyping-by-Sequencing Data
Source: Animals (Basel). 2022 Aug 11;12(16):2052. doi: 10.3390/ani12162052 (PMC9404428; doi:10.3390/ani12162052)
Supplement: Supplementary file 1 [file animals-12-02052-s001.zip › Supplemental Table S1.pdf]

Supplemental Table S1. Quality control statistics for GBS.

Samples	Number of raw reads	Number of clean reads	Proportion of clean reads (%)	Number of raw bases	Number of clean bases	Proportion of clean bases (%)	GC content (%)	Percentage of reads with phred >Q20	Percentage of reads with phred >Q30
SG01	8866756	8627546	97.30	1307846510	1259621716	96.31	50.24	95.72	89.44
SG02	11738720	11319494	96.43	1731461200	1652646124	95.45	51.63	96.48	91.02
SG03	12317750	12001864	97.44	1816868125	1752272144	96.44	51.23	96.52	91.06
SG04	11237510	10876762	96.79	1657532725	1588007252	95.81	51.78	96.39	90.83
SG05	11655482	11326104	97.17	1719183595	1653611184	96.19	51.28	96.30	90.64
SG06	11410562	10963344	96.08	1683057895	1600648224	95.10	52.22	96.49	91.05
SG07	12132486	11713864	96.55	1789541685	1710224144	95.57	51.73	96.53	91.13
SG08	12266168	11789188	96.11	1809259780	1721221448	95.13	52.21	96.51	91.08
SG09	12237434	11848524	96.82	1805021515	1729884504	95.84	51.83	96.40	90.88
SG10	12572730	12214620	97.15	1854477675	1783334520	96.16	51.63	96.31	90.67
SG11	12795362	12396322	96.88	1887315895	1809863012	95.90	51.37	96.46	90.99
SG12	12546198	12089964	96.36	1850564205	1765134744	95.38	51.94	96.41	90.84
SG13	13355714	12985626	97.23	1969967815	1895901396	96.24	51.60	96.34	90.74
SG14	12665066	12182052	96.19	1868097235	1778579592	95.21	51.80	96.52	91.12
SG15	12690632	12295318	96.88	1871868220	1795116428	95.90	51.87	96.63	91.33
SG16	11886016	11440952	96.26	1753187360	1670378992	95.28	52.37	96.53	91.11
SG17	13388320	12818328	95.74	1974777200	1871475888	94.77	51.76	96.33	90.75
SG18	12364926	11886676	96.13	1823826585	1735454696	95.15	51.97	96.50	91.03
SG19	11733914	11358070	96.80	1730752315	1658278220	95.81	51.61	96.45	90.95
SG20	13114394	12617618	96.21	1934373115	1842172228	95.23	52.01	96.61	91.30
SG21	13187304	12744666	96.64	1945127340	1860721236	95.66	51.72	96.49	91.05

SG22	14279932	13724408	96.11	2106289970	2003763568	95.13	51.66	96.31	90.69
SG23	13800658	13222794	95.81	2028696726	1930527924	95.16	52.07	96.53	91.16
SG24	12927012	12218802	94.52	1900270764	1783945092	93.88	52.45	96.45	90.98
SG25	13189614	12812604	97.14	1938873258	1870640184	96.48	51.31	96.22	90.48
SG26	11087258	10628916	95.87	1629826926	1551821736	95.21	52.34	96.56	91.20
SG27	13821216	13389664	96.88	2031718752	1954890944	96.22	51.68	96.32	90.70
SG28	12799076	12379766	96.72	1881464172	1807445836	96.07	51.70	96.36	90.79
SG29	12248460	11872026	96.93	1800523620	1733315796	96.27	51.60	96.36	90.81
SG30	11877570	11411572	96.08	1746002790	1666089512	95.42	52.12	96.54	91.13
SW01	14208570	13717956	96.55	2088659790	2002821576	95.89	51.76	96.54	91.14
SW02	11513450	10981076	95.38	1692477150	1603237096	94.73	52.42	96.45	91.00
SW03	13942914	13419350	96.24	2049608358	1959225100	95.59	51.76	96.49	91.08
SW04	12762982	12296974	96.35	1876158354	1795358204	95.69	51.93	96.50	91.08
SW05	12400734	11966984	96.50	1822907898	1747179664	95.85	52.01	96.60	91.27
SW06	12569642	12150710	96.67	1847737374	1774003660	96.01	52.01	96.39	90.86
SW07	13596598	13095956	96.32	1998699906	1912009576	95.66	51.90	96.55	91.14
SW08	13126920	12735700	97.02	1929657240	1859412200	96.36	51.46	96.40	90.88
SW09	13219906	12825204	97.01	1943326182	1872479784	96.35	51.68	96.41	90.88
SW10	12256072	11865780	96.82	1801642584	1732403880	96.16	51.88	96.31	90.65
SW11	13497466	13032442	96.55	1984127502	1902736532	95.90	52.48	96.59	91.25
SW12	13492880	13037030	96.62	1983453360	1903406380	95.96	52.48	96.40	90.84
SW13	13245728	12744228	96.21	1947122016	1860657288	95.56	52.70	96.73	91.56
SW14	14249338	13743144	96.45	2094652686	2006499024	95.79	52.72	96.70	91.48
SW15	14552068	14076512	96.73	2139153996	2055170752	96.07	52.39	96.55	91.19
SW16	12471708	12094822	96.98	1833341076	1765844012	96.32	51.40	96.31	90.66
SW17	12942894	12509998	96.66	1902605418	1826459708	96.00	52.05	96.41	90.90

SW18	12666610	12186984	96.21	1861991670	1779299664	95.56	52.36	96.59	91.26
SW19	13148322	12674316	96.39	1932803334	1850450136	95.74	51.27	96.36	90.79
SW20	12729412	12312600	96.73	1871223564	1797639600	96.07	51.33	96.35	90.76
SW21	12451438	12016594	96.51	1830361386	1754422724	95.85	51.77	96.23	90.51
SW22	13797396	13370428	96.91	2028217212	1952082488	96.25	51.60	96.50	91.05
SW23	13878002	13363866	96.30	2040066294	1951124436	95.64	51.87	96.43	90.94
SW24	13419250	12917320	96.26	1972629750	1885928720	95.60	51.66	96.33	90.73
SW25	11899796	11526454	96.86	1749270012	1682862284	96.20	51.52	96.49	91.06
SW26	13060970	12564712	96.20	1919962590	1834447952	95.55	51.69	96.21	90.43
SW27	12753858	12235906	95.94	1874817126	1786442276	95.29	51.77	96.54	91.15
SW28	12237278	11738644	95.93	1798879866	1713842024	95.27	52.13	96.57	91.21
SW29	12897226	12416960	96.28	1895892222	1812876160	95.62	51.41	96.41	90.90
SW30	13061492	12537442	95.99	1920039324	1830466532	95.33	52.00	96.40	90.87
CF01	12838828	12385056	96.47	1887307716	1808218176	95.81	51.68	96.49	91.07
CF02	11935334	11497918	96.34	1754494098	1678696028	95.68	51.81	96.53	91.16
CF03	13350746	12917852	96.76	1962559662	1886006392	96.10	51.55	96.54	91.15
CF04	12898124	12457626	96.58	1896024228	1818813396	95.93	51.41	96.54	91.15
CF05	12758118	12331508	96.66	1875443346	1800400168	96.00	51.18	96.39	90.82
CF06	12388960	11839522	95.57	1821177120	1728570212	94.91	52.34	96.46	90.99
CF07	11723368	11170126	95.28	1723335096	1630838396	94.63	51.90	96.28	90.59
CF08	12463720	12001658	96.29	1832166840	1752242068	95.64	51.68	96.27	90.59
CF09	11349334	10976218	96.71	1668352098	1602527828	96.05	51.24	96.30	90.63
CF10	11917432	11463392	96.19	1739945072	1673655232	96.19	51.55	96.09	90.19
CF11	12135892	11758772	96.89	1783976124	1716780712	96.23	51.19	96.15	90.32
CF12	11923770	11466908	96.17	1740870420	1674168568	96.17	51.66	96.33	90.70
CF13	12314556	11766710	95.55	1810239732	1717939660	94.90	51.88	96.54	91.16

CF14	12608120	12096054	95.94	1853393640	1766023884	95.29	51.88	96.42	90.94
CF15	13060894	12611198	96.56	1919951418	1841234908	95.90	51.81	96.45	90.94
CF16	13067548	12566358	96.16	1920929556	1834688268	95.51	52.05	96.45	90.98
CF17	13206876	12801738	96.93	1941410772	1869053748	96.27	51.64	96.50	91.06
CF18	12558946	12168366	96.89	1846165062	1776581436	96.23	51.95	96.29	90.64
CF19	12842550	12306840	95.83	1881433575	1796798640	95.50	52.55	96.41	90.87
CF20	12350104	11901540	96.37	1809290236	1737624840	96.04	52.21	96.41	90.88
CF21	12588218	11965856	95.06	1844173937	1747014976	94.73	52.51	96.52	91.11
CF22	11306376	10660748	94.29	1656384084	1556469208	93.97	52.71	96.42	90.92
CF23	13605928	13166594	96.77	1993268452	1922322724	96.44	51.62	96.46	90.99
CF24	12910650	12295988	95.24	1891410225	1795214248	94.91	52.73	96.59	91.26
CF25	13235326	12712034	96.05	1938975259	1855956964	95.72	52.39	96.37	90.79
CF26	12403674	11881230	95.79	1817138241	1734659580	95.46	52.33	96.40	90.87
CF27	11772172	11256282	95.62	1724623198	1643417172	95.29	52.50	96.64	91.34
CF28	13191880	12671302	96.05	1932610420	1850010092	95.73	52.53	96.50	91.04
CF29	12420976	11891692	95.74	1819672984	1736187032	95.41	51.96	96.38	90.83
CF30	12633060	12163200	96.28	1850743290	1775827200	95.95	51.71	96.38	90.83
QX01	13044784	12537260	96.11	1911060856	1830439960	95.78	52.07	96.47	91.00
QX02	13318900	12750824	95.73	1951218850	1861620304	95.41	52.34	96.23	90.49
QX03	12575002	12186870	96.91	1842237793	1779283020	96.58	51.87	96.21	90.45
QX04	12684328	12083728	95.27	1858254052	1764224288	94.94	52.04	96.16	90.37
QX05	12544016	12130156	96.70	1837698344	1771002776	96.37	51.74	96.19	90.42
QX06	12910336	12400982	96.05	1891364224	1810543372	95.73	52.35	96.31	90.68
QX07	12178540	11593650	95.20	1784156110	1692672900	94.87	53.21	96.51	91.09
QX08	11833758	11203776	94.68	1733645547	1635751296	94.35	52.64	96.28	90.63
QX09	12922194	12314856	95.30	1893101421	1797968976	94.97	52.53	96.55	91.18

QX10	13112760	12561276	95.79	1921019340	1833946296	95.47	52.73	96.39	90.83
QX11	12092448	11724460	96.96	1771543632	1711771160	96.63	52.25	96.10	90.20
QX12	11954504	11628704	97.27	1751334836	1697790784	96.94	52.01	96.32	90.70
QX13	11710610	11427424	97.58	1715604365	1668403904	97.25	51.59	96.25	90.52
QX14	12274950	11956316	97.40	1798280175	1745622136	97.07	51.23	96.07	90.17
QX15	13582000	13173116	96.99	1989763000	1923274936	96.66	51.83	96.28	90.63
QX16	13621736	13156926	96.59	1995584324	1920911196	96.26	51.95	96.38	90.83
QX17	12802266	12424616	97.05	1875531969	1813993936	96.72	51.81	96.34	90.70
QX18	12467360	11917032	95.59	1826468240	1739886672	95.26	52.46	96.50	91.06
QX19	12246672	11814940	96.47	1794137448	1724981240	96.15	52.09	96.27	90.57
QX20	13325092	12830768	96.29	1952125978	1873292128	95.96	51.70	96.34	90.76
QX21	11295938	10832932	95.90	1654854917	1581608072	95.57	52.67	96.14	90.31
QX22	11836590	11135218	94.07	1734060435	1625741828	93.75	53.10	96.59	91.27
QX23	13966184	13466962	96.43	2046045956	1966176452	96.10	51.69	96.26	90.57
QX24	14416054	13920020	96.56	2111951911	2032322920	96.23	51.90	96.44	90.94
QX25	12233682	11661412	95.32	1792234413	1702566152	95.00	52.27	96.41	90.90
QX26	13908784	13426382	96.53	2037636856	1960251772	96.20	52.17	96.12	90.26
QX27	13474292	12996626	96.45	1973983778	1897507396	96.13	52.21	96.29	90.63
QX28	11479670	10961648	95.49	1681771655	1600400608	95.16	52.62	96.31	90.68
QX29	12915216	12507326	96.84	1892079144	1826069596	96.51	51.71	96.52	91.09
QX30	13811948	13296576	96.27	2023450382	1941300096	95.94	52.59	96.31	90.64
ZK01	13303140	12808466	96.28	1948910010	1870036036	95.95	52.26	96.39	90.84
ZK02	12136024	11641870	95.93	1777927516	1699713020	95.60	52.11	96.30	90.65
ZK03	12428624	11994502	96.51	1820793416	1751197292	96.18	52.18	96.28	90.59
ZK04	13061500	12607948	96.53	1913509750	1840760408	96.20	52.48	96.43	90.86
ZK05	12026750	11645890	96.83	1761918875	1700299940	96.50	52.35	95.86	89.62

ZK06	11821758	11463028	96.97	1731887547	1673602088	96.63	51.82	96.11	90.28
ZK07	11657816	11312888	97.04	1707870044	1651681648	96.71	52.19	96.27	90.54
ZK08	13150658	12720948	96.73	1926571397	1857258408	96.40	52.38	96.21	90.50
ZK09	13442522	12865252	95.71	1969329473	1878326792	95.38	52.50	96.41	90.91
ZK10	13221804	12666420	95.80	1936994286	1849297320	95.47	52.04	96.40	90.88
ZK11	12919164	12415934	96.10	1892657526	1812726364	95.78	52.60	96.64	91.33
ZK12	13346688	12863130	96.38	1955289792	1878016980	96.05	52.16	96.48	91.01
ZK13	13681256	13153656	96.14	2004304004	1920433776	95.82	52.38	96.43	90.94
ZK14	13079270	12544278	95.91	1916113055	1831464588	95.58	52.29	96.48	91.07
ZK15	13864316	13329986	96.15	2031122294	1946177956	95.82	52.53	96.53	91.13
ZK16	13393484	12742852	95.14	1962145406	1860456392	94.82	52.97	96.49	91.04
ZK17	12278874	11795966	96.07	1798855041	1722211036	95.74	52.35	96.19	90.42
ZK18	11023852	10685192	96.93	1614994318	1560038032	96.60	51.93	96.23	90.43
ZK19	13686804	13269148	96.95	2005116786	1937295608	96.62	52.31	96.37	90.79
ZK20	13317132	12846266	96.46	1950959838	1875554836	96.13	52.43	96.26	90.56
ZK21	13074940	12619002	96.51	1915478710	1842374292	96.18	52.24	96.17	90.38
ZK22	12818330	12340086	96.27	1877885345	1801652556	95.94	52.42	96.24	90.55
ZK23	12710330	12264798	96.49	1862063345	1790660508	96.17	52.35	96.13	90.27
ZK24	12476864	11947198	95.75	1827860576	1744290908	95.43	52.53	96.48	91.04
ZK25	13600644	13093906	96.27	1992494346	1911710276	95.95	52.48	96.50	91.06
ZK26	13759354	13298080	96.65	2015745361	1941519680	96.32	51.87	96.35	90.77
ZK27	13139880	12606898	95.94	1924992420	1840607108	95.62	53.04	96.52	91.11
ZK28	13568470	13128404	96.76	1987780855	1916746984	96.43	52.10	96.37	90.80
ZK29	13931046	13485642	96.80	2040898239	1968903732	96.47	51.69	96.31	90.67
ZK30	13319368	12685344	95.24	1951287412	1852060224	94.91	52.82	96.51	91.12
