# Supplementary material for: Trait Analysis in Domestic Rabbits (Oryctolagus cuniculus f. domesticus) Using SNP Markers from Genotyping-by-Sequencing Data
Source: Animals (Basel). 2022 Aug 11;12(16):2052. doi: 10.3390/ani12162052 (PMC9404428; doi:10.3390/ani12162052)
Supplement: Supplementary file 1 [file animals-12-02052-s001.zip › Supplemental Table S2.pdf]

Supplemental Table S2. Mapping rate statistics for GBS.

Sample	Mapping rate (%)	SNPs with coverage ≥ 1× (%)	SNPs with coverage ≥ 5× (%)	SNPs with coverage ≥ 10× (%)
SG01	98.83	7.14	3.34	1.50
SG02	98.75	8.30	4.14	2.12
SG03	98.78	8.58	4.40	2.31
SG06	98.72	8.66	4.10	2
SG07	98.75	8.78	4.29	2.20
SG08	98.80	9.16	4.41	2.19
SG09	98.76	8.74	4.33	2.23
SG10	98.70	8.31	4.35	2.37
SG11	98.74	8.33	4.37	2.44
SG12	98.76	8.28	4.23	2.33
SG13	98.72	8.91	4.53	2.52
SG14	98.74	7.83	4.22	2.42
SG15	98.74	8.56	4.37	2.36
SG16	98.76	9.08	4.28	2.07
SG17	98.77	8.39	4.38	2.51
SG18	98.73	8.22	4.18	2.29
SG19	98.76	8.38	4.16	2.19
SG20	98.73	8.45	4.37	2.47
SG21	98.77	8.40	4.42	2.52
SG22	98.73	8.50	4.62	2.78
SG23	98.75	8.63	4.50	2.57
SG24	98.86	8.79	4.33	2.31
SG25	98.74	8.89	4.50	2.51
SG26	98.81	8.68	4	1.92
SG27	98.72	8.89	4.61	2.66
SG28	98.69	8.81	4.40	2.42
SG29	98.73	8.44	4.25	2.33
SG30	98.75	8.80	4.19	2.13
SG04	98.68	8.71	4.10	1.95
SG05	98.70	8.36	4.17	2.14
SW1	98.74	8.84	4.58	2.66
SW02	98.73	9	4.09	1.97
SW03	98.76	8.49	4.49	2.60
SW04	98.74	8.34	4.30	2.38
SW05	98.73	8.51	4.21	2.28
SW06	98.67	8.60	4.32	2.32
SW07	98.76	8.36	4.43	2.56
SW08	98.73	8.71	4.47	2.47
SW09	98.74	8.36	4.41	2.50
SW10	98.72	8.87	4.36	2.24

SW11	98.70	8.78	4.30	2.40
SW12	98.62	8.05	4.29	2.45
SW13	98.69	7.71	3.95	2.39
SW14	98.72	7.90	4.22	2.62
SW15	98.66	8.57	4.50	2.68
SW16	98.75	8.68	4.32	2.24
SW17	98.61	9.22	4.37	2.35
SW18	98.72	9.10	4.34	2.27
SW19	98.73	8.60	4.37	2.45
SW20	98.75	8.39	4.35	2.44
SW21	98.74	9.10	4.36	2.26
SW22	98.76	9.14	4.67	2.59
SW23	98.74	9.08	4.63	2.58
SW24	98.77	8.99	4.53	2.49
SW25	98.74	8.58	4.19	2.19
SW26	98.76	8.94	4.50	2.44
SW27	98.74	8.49	4.26	2.33
SW28	98.81	8.65	4.18	2.17
SW29	98.76	8.36	4.35	2.45
SW30	98.76	8.78	4.41	2.41
CF01	98.74	8.60	4.35	2.40
CF02	98.74	8.52	4.18	2.16
CF03	98.73	8.87	4.40	2.46
CF04	98.70	8.58	4.25	2.34
CF05	98.66	8.13	4.30	2.44
CF06	98.75	9.05	4.36	2.21
CF07	98.71	8.58	4.18	2.10
CF08	98.70	8.57	4.33	2.31
CF09	98.69	8.62	4.17	2.04
CF10	98.68	8.76	4.31	2.13
CF11	98.64	8.58	4.24	2.18
CF12	98.72	8.55	4.25	2.15
CF13	98.71	8.09	4.17	2.28
CF14	98.70	8.32	4.21	2.32
CF15	98.73	8.32	4.36	2.47
CF16	98.69	8.74	4.41	2.41
CF17	98.73	8.51	4.45	2.52
CF18	98.74	8.97	4.44	2.32
CF19	98.77	9.17	4.51	2.31
CF20	98.75	9.10	4.40	2.22
CF21	98.79	9.08	4.40	2.24
CF22	98.82	8.67	4.03	1.90
CF23	98.72	8.75	4.51	2.56
CF24	98.77	9.03	4.40	2.30

CF25	98.77	8.94	4.49	2.41
CF26	98.71	8.92	4.31	2.23
CF27	98.81	8.86	4.17	2.06
CF28	98.79	8.97	4.44	2.39
CF29	98.77	8.78	4.32	2.25
CF30	98.70	8.60	4.32	2.33
QX01	98.72	8.63	4.40	2.39
QX02	98.74	9.26	4.65	2.43
QX03	98.74	8.96	4.53	2.33
QX04	98.76	9.13	4.51	2.28
QX05	98.67	8.89	4.42	2.31
QX06	98.76	9.21	4.53	2.32
QX07	98.63	9.61	4.29	2.03
QX08	98.63	8.84	4.20	2.04
QX09	98.69	8.80	4.38	2.33
QX10	98.68	9.15	4.44	2.34
QX11	98.62	9.26	4.31	2.14
QX12	98.65	9.03	4.26	2.14
QX13	98.66	8.92	4.25	2.11
QX14	98.64	8.71	4.37	2.25
QX15	98.61	8.67	4.50	2.56
QX16	98.69	8.90	4.51	2.54
QX17	98.69	9.05	4.46	2.38
QX18	98.68	8.23	4.28	2.32
QX19	98.78	9.27	4.50	2.20
QX20	98.69	8.45	4.48	2.56
QX21	98.70	8.98	4.11	1.93
QX22	98.79	8.88	4.16	1.99
QX23	98.71	8.61	4.54	2.68
QX24	98.67	8.93	4.69	2.74
QX25	98.77	9.03	4.34	2.20
QX26	98.62	9	4.60	2.60
QX27	98.65	9.30	4.55	2.47
QX28	98.75	9.77	4.26	1.85
QX29	98.68	8.59	4.40	2.45
QX30	98.73	9.53	4.70	2.52
ZK01	98.63	8.89	4.48	2.46
ZK02	98.63	8.75	4.20	2.18
ZK03	98.60	9.49	4.42	2.19
ZK04	98.56	9.50	4.46	2.33
ZK05	98.51	10.13	4.38	2
ZK06	98.54	9.96	4.29	1.99
ZK07	98.55	10.11	4.29	1.91
ZK08	98.54	10.37	4.59	2.24

ZK09	98.68	8.89	4.46	2.44
ZK10	98.73	8.30	4.32	2.46
ZK11	98.69	8.86	4.36	2.31
ZK12	98.68	8.46	4.38	2.48
ZK13	98.67	8.79	4.51	2.51
ZK14	98.74	8.70	4.35	2.37
ZK15	98.68	8.98	4.50	2.52
ZK16	98.78	9.09	4.49	2.39
ZK17	98.70	9.41	4.41	2.18
ZK18	98.73	9.31	4.18	1.87
ZK19	98.62	9.72	4.65	2.50
ZK20	98.70	9.49	4.56	2.43
ZK21	98.69	9.55	4.54	2.39
ZK22	98.68	9.49	4.45	2.32
ZK23	98.67	9.66	4.55	2.28
ZK24	98.74	8.83	4.34	2.25
ZK25	98.78	9.04	4.60	2.51
ZK26	98.75	8.94	4.59	2.54
ZK27	98.75	9.31	4.48	2.33
ZK28	98.66	8.88	4.55	2.53
ZK29	98.75	8.57	4.58	2.68
ZK30	98.78	8.89	4.43	2.40
