# Supplementary material for: Trait Analysis in Domestic Rabbits (Oryctolagus cuniculus f. domesticus) Using SNP Markers from Genotyping-by-Sequencing Data
Source: Animals (Basel). 2022 Aug 11;12(16):2052. doi: 10.3390/ani12162052 (PMC9404428; doi:10.3390/ani12162052)
Supplement: Supplementary file 1 [file animals-12-02052-s001.zip › Supplemental Table S4.pdf]

Supplemental Table S4. Summary result of nucleotide substitution types at SNP loci

	Transitions		Transversions			
Type of SNP	A/G	C/T	A/C	A/T	C/G	G/T
Number of sites	308583	309196	57252	40539	58692	56773
Frequencies	0.371	0.372	0.069	0.049	0.071	0.068
Total	617779 (74.3%)		213256 (25.7%)			
