# Supplementary material for: Trait Analysis in Domestic Rabbits (Oryctolagus cuniculus f. domesticus) Using SNP Markers from Genotyping-by-Sequencing Data
Source: Animals (Basel). 2022 Aug 11;12(16):2052. doi: 10.3390/ani12162052 (PMC9404428; doi:10.3390/ani12162052)
Supplement: Supplementary file 1 [file animals-12-02052-s001.zip › Supplemental Table S5.pdf]

Supplemental Table S5. Summary result of the potential functional effect of SNP variants on gene products

Degree of influence of SNP	Count	Percent
High	256	0.02%
Moderate	8556	0.55%
Low	15191	0.98%
Modifier	1529925	98.46%

High: High (destructive) effect on protein, may cause protein truncation, loss of function, etc. Low: Low impact, probably not affecting protein. Moderate: Moderate impact, non-destructive variation, may affect protein efficacy. Modifier: A modification, usually a noncoding region variant, which affects only noncoding genes, so it is difficult to predict the extent of their impact on the protein. Count: Number of effects.
