# Supplementary material for: Trait Analysis in Domestic Rabbits (Oryctolagus cuniculus f. domesticus) Using SNP Markers from Genotyping-by-Sequencing Data
Source: Animals (Basel). 2022 Aug 11;12(16):2052. doi: 10.3390/ani12162052 (PMC9404428; doi:10.3390/ani12162052)
Supplement: Supplementary file 1 [file animals-12-02052-s001.zip › Supplemental Table S6.pdf]

Supplemental Table S6. Pairwise comparison of genetic differentiation and genetic distances among rabbit breeds

pop	SG	SW	CF	QX	ZK
SG	-	0.0934	0.2408	0.2248	0.2675
SW	0.08913	-	0.1629	0.1526	0.1867
CF	0.214	0.1503	-	0.1057	0.0778
QX	0.2013	0.1415	0.1003	-	0.1381
ZK	0.2347	0.1703	0.07486	0.129	-

The below the diagonal is the co-efficient of genetic differentiation between populations (Fst), and above the diagonal we listed the pairwise genetic distances among breeds. CF, Californian rabbit; ZK, German Zika rabbit; QX, Qixing rabbit; SG, Sichuan grey rabbit; SW, Sichuan white rabbit.
