# Supplementary material for: Trait Analysis in Domestic Rabbits (Oryctolagus cuniculus f. domesticus) Using SNP Markers from Genotyping-by-Sequencing Data
Source: Animals (Basel). 2022 Aug 11;12(16):2052. doi: 10.3390/ani12162052 (PMC9404428; doi:10.3390/ani12162052)
Supplement: Supplementary file 1 [file animals-12-02052-s001.zip › Supplementary Figure S1.pdf]

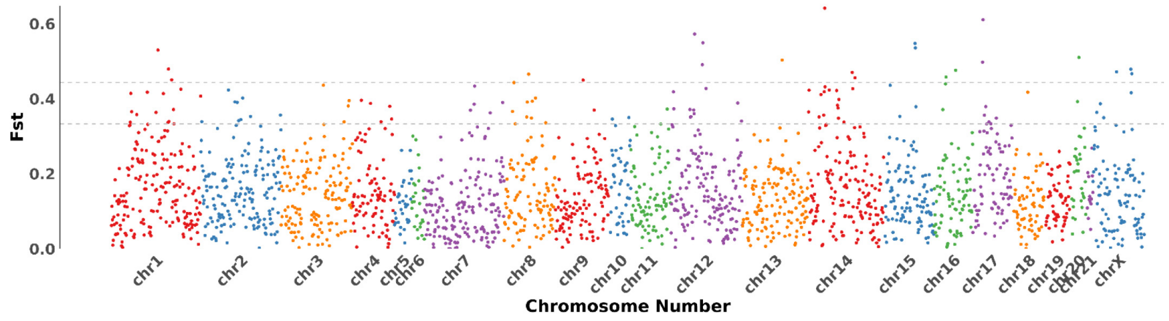

SW vs CF

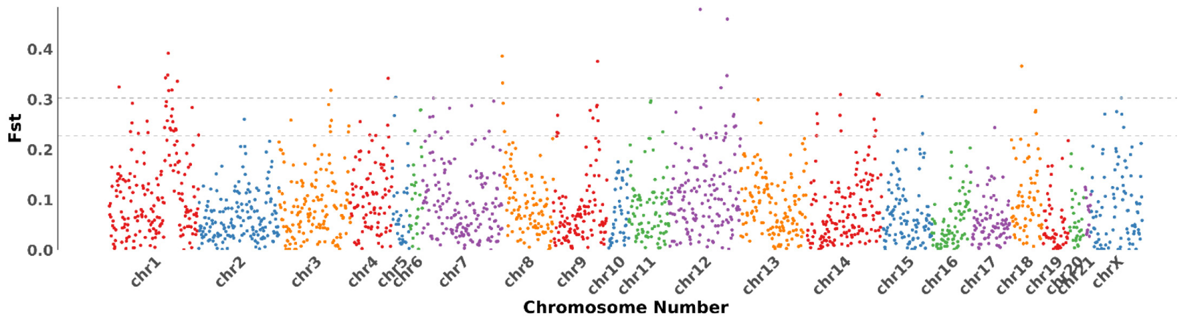

SW vs SG

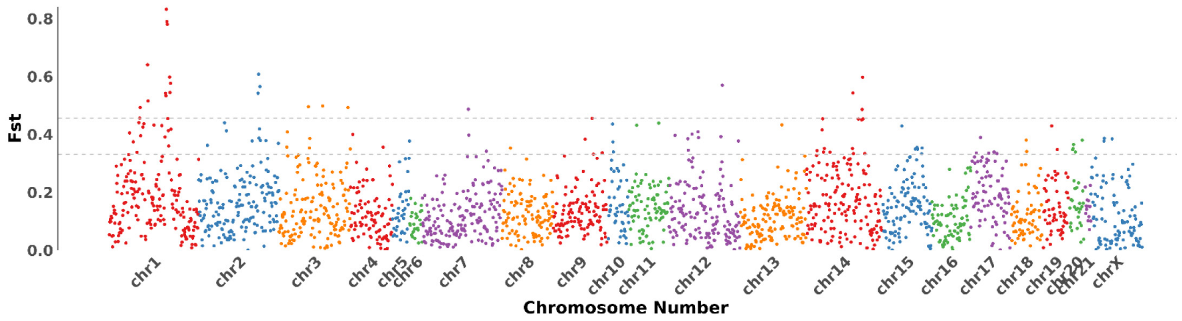

SW vs QX

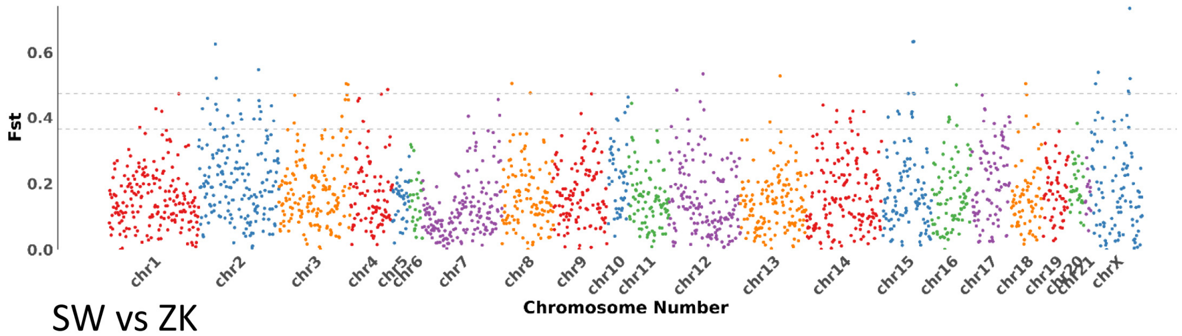

SW vs ZK

Supplementary Figure S1. Coefficient (Fst) of genetic differentiation between breeds. The abscissa represents different chromosome names, the ordinate represents the Fst value in the corresponding chromosome window, and the two dotted lines represent the two selection thresholds (top 5% or 1%).
